# 4-Phenyl-2,6-bis­(4-tol­yl)pyridine

**DOI:** 10.1107/S1600536810031764

**Published:** 2010-08-18

**Authors:** Yajun Ma, Buming Liu, Chenghu Xue

**Affiliations:** a School of Chemistry and Chemical Engineering, Yu Lin University, Yulin 719000, People’s Republic of China

## Abstract

The title mol­ecule, C_25_H_21_N, situated on the crystallographic twofold axis has a symmetry point group 2. The inter­planar angles between the central pyridyl ring and the phenyl and the methyl­phenyl rings are 32.8 (2) and 23.7 (2)°, respectively. In the crystal packing, the central pyridyl rings of adjacent mol­ecules are involved in π–π inter­actions, forming one-dimensional arrays along the *c* axis with centroid–centroid distances of 3.714 (1) Å.

## Related literature

For the synthesis of Kröhnke-type pyridines, see: Cave & Raston (2001 ▶); Kröhnke (1976 ▶).
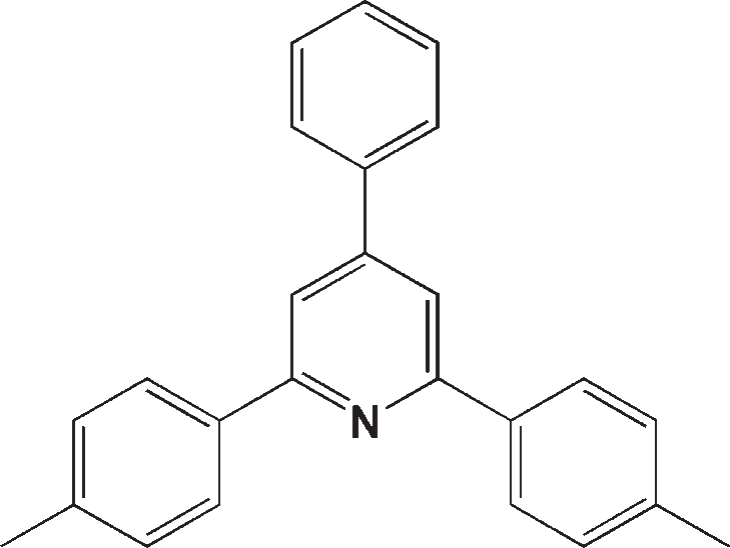


## Experimental

### 

#### Crystal data

C_25_H_21_N
*M* *_r_* = 335.43
Orthorhombic, 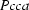

*a* = 21.234 (3) Å
*b* = 12.0489 (15) Å
*c* = 7.3601 (10) Å
*V* = 1883.1 (4) Å^3^
*Z* = 4
Mo *K*α radiation
μ = 0.07 mm^−1^
*T* = 295 K
0.24 × 0.16 × 0.14 mm

#### Data collection

Bruker SMART APEX area-detector diffractometer
Absorption correction: multi-scan (*SADABS*; Sheldrick, 1996 ▶) *T*_min_ = 0.984, *T*_max_ = 0.991
6295 measured reflections
1833 independent reflections
1130 reflections with *I* > 2σ(*I*)
*R*_int_ = 0.030

#### Refinement

*R*[*F* ^2^ > 2σ(*F* ^2^)] = 0.058
*wR*(*F* ^2^) = 0.179
*S* = 1.02
1833 reflections
121 parameters
H-atom parameters constrained
Δρ_max_ = 0.13 e Å^−3^
Δρ_min_ = −0.12 e Å^−3^

### 

Data collection: *SMART* (Bruker, 2002 ▶); cell refinement: *SAINT* (Bruker, 2002 ▶); data reduction: *SAINT*; program(s) used to solve structure: *SHELXS97* (Sheldrick, 2008 ▶); program(s) used to refine structure: *SHELXL97* (Sheldrick, 2008 ▶); molecular graphics: *SHELXTL* (Sheldrick, 2008 ▶); software used to prepare material for publication: *SHELXTL* and *PLATON* (Spek, 2009 ▶).

## Supplementary Material

Crystal structure: contains datablocks I, global. DOI: 10.1107/S1600536810031764/fb2207sup1.cif

Structure factors: contains datablocks I. DOI: 10.1107/S1600536810031764/fb2207Isup2.hkl

Additional supplementary materials:  crystallographic information; 3D view; checkCIF report

## Figures and Tables

**Fig. 1.**
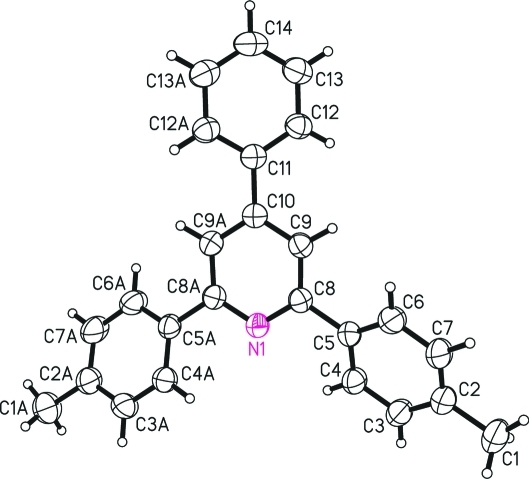
The title molecule with displacement ellipsoids drawn at the 30% probability level. The H atoms are shown as spheres of arbitrary radii. The atoms labelled by "A" are related to their counterparts by the rotation by 180° about the crystallographic two-fold axis.

**Fig. 2.**
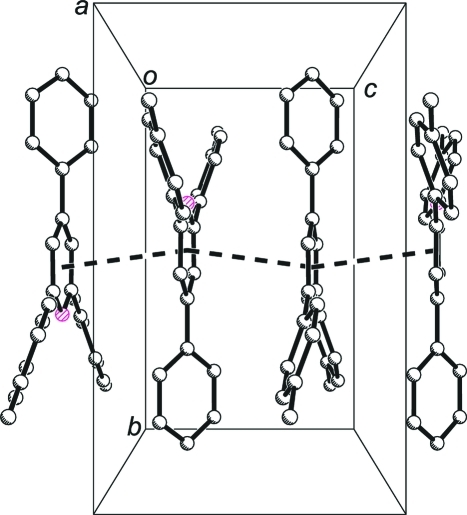
Packing diagram of the title compound showing the intermolecular π-electron ring···π-electron ring interactions as dashed lines. The H atoms have been omitted for clarity.

## supplementary crystallographic information

### Comment

The Kröhnke type pyridines with different substituents as well as their syntheses have been widely studied. The reason is a prominent functionalization of the Kröhnke type pyridines as building blocks in both organic and inorganic supramolecular chemistry (Cave & Raston, 2001; Kröhnke, 1976). In this article,  the synthesis and the crystal structure of a new Kröhnke type pyridine compound, 4-phenyl-2,6-bis-(4-tolyl)-pyridine, is presented.

The title molecule shows symmetry 2. The two-fold axis passes through the central pyridine N1, C10, C11, C14 and H14 atoms (Fig. 1). The interplanar angle between central pyridyl ring (N1—C10) and the phenyl ring (C11—C14) is 32.8 (2)°, while the interplanar angle between the central pyridyl ring and methylphenyl ring (C2—C7) equals to 23.7 (2)°. The central pyridyl rings of the adjacent molecules are connected by intermolecular π-electron ring···π-electron ring interactions to form one-dimensional arrays along the *c* axis. The pertinent centroid-to-centroid distances equal to 3.714 (1) Å (Fig. 2). The centroid coordinates are 0.00000 (3), 0.52074 (7), 0.25000 (7) (Spek, 2009).

### Experimental

The mixture of benzaldehyde (1.06 g, 10 mmol), 4-methylacetophenone (2.68 g, 20 mmol) and NaOH (0.80 g, 20 mmol) in water (20 ml) and 95% ethanol (20 ml) was stirred for 3 h at room temperature, then the solution of ammonium acetate (7.70 g, 100 mmol) in 95% ethanol (60 ml) was added, and further refluxed at 343 K for 8 h. The resulting solution was cooled, solvent reduced to 20 ml to give a white precipitate which was collected by filtration and washed with ethanol. Recrystallization from 95% ethanol gave colorless prism crystals of the title compound with sizes of about 2.0 × 0.5 × 0.1 mm. Yield: 0.41 g (12%).

### Refinement

All the hydrogens were observable in the difference electron density map. However, they were placed into the idealized  positions and refined using a riding atom formalism. C-H_aryl_=0.93Å, C-H_methyl_=0.96 Å. *U*_iso_(H_aryl_)=1.2*U*_eq_(C_aryl_); *U*_iso_(H_methyl_)=1.5*U*_eq_(C_methyl_).

### Crystal data

**Table:**

C_25_H_21_N	*F*(000) = 712
*M**_r_* = 335.43	*D*_x_ = 1.183 Mg m^−^^3^
Orthorhombic, *P**c**c**a*	Mo *K*α radiation, λ = 0.71073 Å
Hall symbol: -P 2a 2ac	Cell parameters from 1019 reflections
*a* = 21.234 (3) Å	θ = 2.6–23.3°
*b* = 12.0489 (15) Å	µ = 0.07 mm^−^^1^
*c* = 7.3601 (10) Å	*T* = 295 K
*V* = 1883.1 (4) Å^3^	Prism, colourless
*Z* = 4	0.24 × 0.16 × 0.14 mm

### Data collection

**Table:**

Bruker SMART APEX area-detector diffractometer	1833 independent reflections
Radiation source: fine-focus sealed tube	1130 reflections with *I* > 2σ(*I*)
graphite	*R*_int_ = 0.030
φ and ω scans	θ_max_ = 26.0°, θ_min_ = 2.6°
Absorption correction: multi-scan (*SADABS*; Sheldrick, 1996)	*h* = −26→26
*T*_min_ = 0.984, *T*_max_ = 0.991	*k* = −14→12
6295 measured reflections	*l* = −9→2

### Refinement

**Table:**

Refinement on *F*^2^	Primary atom site location: structure-invariant direct methods
Least-squares matrix: full	Secondary atom site location: difference Fourier map
*R*[*F*^2^ > 2σ(*F*^2^)] = 0.058	Hydrogen site location: difference Fourier map
*wR*(*F*^2^) = 0.179	H-atom parameters constrained
*S* = 1.02	*w* = 1/[σ^2^(*F*_o_^2^) + (0.091*P*)^2^ + 0.1078*P*] where *P* = (*F*_o_^2^ + 2*F*_c_^2^)/3
1833 reflections	(Δ/σ)_max_ < 0.001
121 parameters	Δρ_max_ = 0.13 e Å^−^^3^
0 restraints	Δρ_min_ = −0.12 e Å^−^^3^
41 constraints	

### Special details

**Table:**

Geometry. All esds (except the esd in the dihedral angle between two l.s. planes) are estimated using the full covariance matrix. The cell esds are taken into account individually in the estimation of esds in distances, angles and torsion angles; correlations between esds in cell parameters are only used when they are defined by crystal symmetry. An approximate (isotropic) treatment of cell esds is used for estimating esds involving l.s. planes.
Refinement. Refinement of F^2^ against ALL reflections. The weighted R-factor wR and goodness of fit S are based on F^2^, conventional R-factors R are based on F, with F set to zero for negative F^2^. The threshold expression of F^2^ > 2sigma(F^2^) is used only for calculating R-factors(gt) etc. and is not relevant to the choice of reflections for refinement. R-factors based on F^2^ are statistically about twice as large as those based on F, and R- factors based on ALL data will be even larger.

### Fractional atomic coordinates and isotropic or equivalent isotropic displacement parameters (Å^2^)

**Table:**

	*x*	*y*	*z*	*U*_iso_*/*U*_eq_	
N1	0.0000	0.63606 (17)	0.2500	0.0650 (6)	
C1	0.28100 (10)	0.8434 (2)	0.3583 (5)	0.1274 (12)	
H1A	0.3171	0.7958	0.3474	0.191*	
H1B	0.2811	0.8780	0.4758	0.191*	
H1C	0.2826	0.8994	0.2657	0.191*	
C2	0.22150 (10)	0.7754 (2)	0.3362 (4)	0.0965 (8)	
C3	0.16260 (9)	0.81731 (18)	0.3759 (4)	0.0895 (8)	
H3	0.1591	0.8899	0.4179	0.107*	
C4	0.10883 (9)	0.75416 (17)	0.3549 (3)	0.0778 (6)	
H4	0.0699	0.7847	0.3836	0.093*	
C5	0.11203 (8)	0.64629 (16)	0.2921 (3)	0.0686 (6)	
C6	0.17095 (9)	0.6050 (2)	0.2485 (4)	0.0993 (9)	
H6	0.1747	0.5331	0.2036	0.119*	
C7	0.22435 (10)	0.6695 (2)	0.2710 (5)	0.1153 (11)	
H7	0.2634	0.6397	0.2407	0.138*	
C8	0.05366 (8)	0.57880 (17)	0.2701 (2)	0.0637 (5)	
C9	0.05510 (8)	0.46379 (17)	0.2715 (2)	0.0670 (6)	
H9	0.0932	0.4270	0.2870	0.080*	
C10	0.0000	0.4032 (2)	0.2500	0.0640 (7)	
C11	0.0000	0.2803 (2)	0.2500	0.0657 (7)	
C12	0.04268 (9)	0.22100 (17)	0.3546 (3)	0.0748 (6)	
H12	0.0718	0.2590	0.4257	0.090*	
C13	0.04258 (10)	0.10657 (18)	0.3546 (3)	0.0882 (7)	
H13	0.0715	0.0682	0.4257	0.106*	
C14	0.0000	0.0489 (3)	0.2500	0.0930 (10)	
H14	0.0000	−0.0283	0.2500	0.112*	

### Atomic displacement parameters (Å^2^)

**Table:**

	*U*^11^	*U*^22^	*U*^33^	*U*^12^	*U*^13^	*U*^23^
N1	0.0574 (12)	0.0664 (14)	0.0710 (15)	0.000	−0.0003 (10)	0.000
C1	0.0739 (15)	0.116 (2)	0.193 (4)	−0.0204 (13)	−0.0066 (18)	−0.0032 (19)
C2	0.0660 (14)	0.0832 (17)	0.140 (2)	−0.0064 (12)	−0.0059 (13)	0.0046 (15)
C3	0.0723 (15)	0.0760 (14)	0.120 (2)	−0.0075 (11)	0.0012 (12)	−0.0090 (13)
C4	0.0614 (12)	0.0758 (14)	0.0961 (15)	0.0008 (10)	0.0040 (10)	−0.0063 (12)
C5	0.0567 (11)	0.0685 (12)	0.0805 (13)	0.0009 (9)	−0.0014 (9)	0.0057 (10)
C6	0.0675 (14)	0.0712 (14)	0.159 (3)	0.0068 (11)	0.0054 (13)	−0.0035 (15)
C7	0.0545 (13)	0.0880 (18)	0.203 (3)	0.0053 (11)	0.0060 (15)	0.0033 (18)
C8	0.0615 (11)	0.0672 (13)	0.0624 (12)	0.0011 (9)	0.0022 (8)	0.0008 (9)
C9	0.0631 (12)	0.0688 (13)	0.0692 (12)	0.0039 (8)	−0.0005 (8)	0.0019 (10)
C10	0.0687 (16)	0.0654 (17)	0.0578 (16)	0.000	0.0033 (12)	0.000
C11	0.0651 (16)	0.0657 (17)	0.0664 (17)	0.000	0.0108 (12)	0.000
C12	0.0748 (13)	0.0701 (13)	0.0795 (14)	0.0034 (10)	0.0063 (10)	0.0007 (11)
C13	0.0885 (15)	0.0759 (15)	0.1001 (19)	0.0089 (12)	0.0109 (12)	0.0086 (13)
C14	0.105 (2)	0.0607 (18)	0.114 (3)	0.000	0.025 (2)	0.000

### Geometric parameters (Å, °)

**Table:**

N1—C8	1.340 (2)	C6—H6	0.9300
N1—C8^i^	1.340 (2)	C7—H7	0.9300
C1—C2	1.515 (3)	C8—C9	1.386 (3)
C1—H1A	0.9600	C9—C10	1.388 (2)
C1—H1B	0.9600	C9—H9	0.9300
C1—H1C	0.9600	C10—C9^i^	1.388 (2)
C2—C7	1.365 (3)	C10—C11	1.480 (4)
C2—C3	1.380 (3)	C11—C12	1.387 (2)
C3—C4	1.381 (3)	C11—C12^i^	1.387 (2)
C3—H3	0.9300	C12—C13	1.379 (3)
C4—C5	1.381 (3)	C12—H12	0.9300
C4—H4	0.9300	C13—C14	1.376 (3)
C5—C6	1.384 (3)	C13—H13	0.9300
C5—C8	1.491 (2)	C14—C13^i^	1.376 (3)
C6—C7	1.384 (3)	C14—H14	0.9300
			
C8—N1—C8^i^	118.0 (2)	C2—C7—H7	119.0
C2—C1—H1A	109.5	C6—C7—H7	119.0
C2—C1—H1B	109.5	N1—C8—C9	122.30 (17)
H1A—C1—H1B	109.5	N1—C8—C5	115.96 (18)
C2—C1—H1C	109.5	C9—C8—C5	121.73 (16)
H1A—C1—H1C	109.5	C8—C9—C10	120.43 (18)
H1B—C1—H1C	109.5	C8—C9—H9	119.8
C7—C2—C3	117.2 (2)	C10—C9—H9	119.8
C7—C2—C1	120.4 (2)	C9—C10—C9^i^	116.5 (2)
C3—C2—C1	122.3 (2)	C9—C10—C11	121.74 (12)
C2—C3—C4	121.6 (2)	C9^i^—C10—C11	121.74 (12)
C2—C3—H3	119.2	C12—C11—C12^i^	118.0 (3)
C4—C3—H3	119.2	C12—C11—C10	121.01 (13)
C3—C4—C5	121.02 (19)	C12^i^—C11—C10	121.01 (13)
C3—C4—H4	119.5	C13—C12—C11	120.9 (2)
C5—C4—H4	119.5	C13—C12—H12	119.5
C4—C5—C6	117.42 (19)	C11—C12—H12	119.5
C4—C5—C8	120.58 (17)	C14—C13—C12	120.4 (2)
C6—C5—C8	122.0 (2)	C14—C13—H13	119.8
C5—C6—C7	120.7 (2)	C12—C13—H13	119.8
C5—C6—H6	119.6	C13—C14—C13^i^	119.3 (3)
C7—C6—H6	119.6	C13—C14—H14	120.4
C2—C7—C6	122.0 (2)	C13^i^—C14—H14	120.4
			
C7—C2—C3—C4	1.5 (4)	C4—C5—C8—C9	−156.5 (2)
C1—C2—C3—C4	180.0 (2)	C6—C5—C8—C9	24.5 (3)
C2—C3—C4—C5	−0.4 (4)	N1—C8—C9—C10	0.7 (2)
C3—C4—C5—C6	−1.0 (3)	C5—C8—C9—C10	−179.76 (15)
C3—C4—C5—C8	179.96 (19)	C8—C9—C10—C9^i^	−0.31 (12)
C4—C5—C6—C7	1.2 (4)	C8—C9—C10—C11	179.69 (12)
C8—C5—C6—C7	−179.8 (2)	C9—C10—C11—C12	32.62 (13)
C3—C2—C7—C6	−1.3 (4)	C9^i^—C10—C11—C12	−147.38 (13)
C1—C2—C7—C6	−179.8 (3)	C9—C10—C11—C12^i^	−147.38 (13)
C5—C6—C7—C2	0.0 (5)	C9^i^—C10—C11—C12^i^	32.62 (12)
C8^i^—N1—C8—C9	−0.33 (12)	C12^i^—C11—C12—C13	−0.07 (14)
C8^i^—N1—C8—C5	−179.93 (18)	C10—C11—C12—C13	179.93 (14)
C4—C5—C8—N1	23.1 (3)	C11—C12—C13—C14	0.1 (3)
C6—C5—C8—N1	−155.9 (2)	C12—C13—C14—C13^i^	−0.07 (14)

Symmetry codes: (i) −*x*, *y*, −*z*+1/2.
